# Precautions to Be Observed in Administration of Milk in Disease

**Published:** 1909-03

**Authors:** J. Michell Clarke

**Affiliations:** Physician to the Bristol General Hospital


### PRECAUTIONS TO BE OBSERVED IN ADMINISTRATION OF MILK IN DISEASE.

BY

J. Michell Clarke, M.A., M.D. Cantab., F.R.C.P.,

*Physician to the Bristol General Hospital.*

(*The following is an abstract of Dr. Clarke's remarks.*)

Before dealing very briefly with special diseases, a few general points about milk require to be borne in mind. Speaking generally, it is perhaps the most universally valuable nutriment in disease, and is most suitable wherever rapid growth or repair of tissue is taking place, as in children, young adults, and in convalescence from acute disease.

As compared with other proteid-containing foods, it calls out the smallest amount of secretion of gastric juice. The pancreatic juice secreted on a milk diet shows, however, a very high proteoclastic power ; hence the digestion of milk proteids takes place chiefly in the intestine, and the work of the stomach is spared. The proteid of milk is readily assimilated, and more completely used up than any other form. The proportion of fat is large, and in a state of emulsion, which is partly the reason why milk is especially valuable in wasting diseases and some nervous affections. Putrefactive processes in the intestines are reduced, as is also the quantity of uric acid excreted. On the other hand, some persons have such an invincible repugnance to or distaste for milk, that they find it impossible to take it.

On a pure milk diet, to obtain the requisite amount of calories a very large quantity of fluid must be taken. Three pints of milk, generally regarded as the minimum daily quantity, only contains sixty to seventy grammes of proteid, and this is not a sufficient amount for more than two or three weeks. To obtain the full amount of calories needful, five to six pints of milk must be taken daily. Besides the inconvenience of taking such a quantity of fluid, there is an excessive flow of urine, and in many persons uncomfortable peristaltic movements of the intestines. In the gastric digestion of milk, the dense and tough clots formed are often difficult of digestion, and cause epigastric discomfort or pain. Constipation is a well-known consequence of a milk diet in many persons. Two other drawbacks to milk are its low content of chlorides and of iron. From the former may arise disturbance in the osmotic processes of the body, and from the latter anæmia.

To turn to the drawbacks to the use of milk in gastric and intestinal affections. It must first be remembered that milk is not strictly speaking a fluid food, that it forms peculiarly dense masses of tough curd in the stomach, and that these are largely carried on into the intestines. Even when milk-digestion is well carried out—and in many persons this is a difficult matter—the fæces on a pure milk diet are bulky, and if large quantities are taken there is a probability that they will not all be properly dealt with, and that undigested curds will be passed in the fæces. In every case, therefore, in which a patient is on a pure milk diet the fæces should be frequently inspected.

In many cases of gastritis, or various forms of neurotic dyspepsia, a course of milk diet gives excellent results ; but in most it is advisable to add to the milk something to render the precipitated curds more flocculent, such as citrate of soda, lime-water or barley-water, or it may be peptonised. In cases of this class the important factor in the cure is that milk makes so little demand upon the gastric glands. In cases of gastric ulcer milk alone as the staple diet, even when one of the above adjuvants is added, is not so satisfactory. Recent observations have shown that it is difficult to maintain nutrition adequately on the reduced amount of milk that such patients can, as a rule, take without discomfort ; consequently that the ulcer does not heal quickly, pain and other symptoms persist, and convalescence is protracted. Further, the large amount of fluid on a purely milk diet is disadvantageous by distending the stomach. A dietary containing more fat and proteid and less water, such as Lenhart's, conduces to a more speedy recovery ; the nutrition is well maintained, the patients put on weight, and the protracted convalescence so often seen in cases of gastric ulcer treated for weeks on a pure milk diet is abolished. The fat in milk, being an emulsion, has probably no effect in reducing the excessive secretion of HCl generally present in ulcer.

Diseases of the stomach attended by dilatation, from whatever cause the pyloric obstruction may arise, are also not suitable for a pure milk diet, although they may do well on small amounts of milk for a few days in beginning a course of treatment, because such stomachs are unable to dispose of the large quantity of fluid involved.

Similar objections apply to the use of milk alone in enteric fever. In the first place, something should always be added to the milk to render the curds more flocculent ; and, secondly, it should be remembered that there is a more bulky residue in the intestines from milk than from many other forms of proteid-containing food. Then again it is impossible to adequately maintain the patient's nutrition on milk only through a long illness such as enteric fever. In enteric fever the milk curds may escape digestion, undergo putrefactive changes in the intestines, giving rise to excessive tympanites, or even to the absorption of toxic matters. Whenever there is much tympanites in enteric fever, the digestion of the milk used should be looked to, and it will often require to be peptonised. For many years I have given other articles of diet from the beginning of the illness in enteric fever, and I think that the patients have in consequence made a better and more speedy recovery, and had a less protracted convalescence. Whether they have shown less tendency to thrombosis of veins I cannot positively say. In convalescence milk can be given freely in addition to the other food, and is generally well digested.

In acute nephritis an absolute milk diet is undesirable because of (1) its high proteid content, and (2) the large amount of fluid involved to obtain a sufficient quantity of nutriment. Three pints of milk per diem is generally regarded as the minimum amount, and this contains sixty to seventy grammes of proteid, an insufficient quantity for the daily needs for more than two to three weeks, and too much water. Later, when the case has passed into a less acute stage, and the kidney begins to secrete water well, more milk can be given. Von Noorden, therefore, recommends the addition of cream in order to obtain the requisite number of calories from a smaller amount of milk. He also advises the addition of calcium carbonate to combine with the phosphoric acid of milk, which is badly excreted by the kidneys.

Albuminuria is not to be regarded as an indication for a pure milk diet. Albuminuria is in some cases a sign rather of past damage to the kidney than of actual and increasing mischief, and in such patients a mixed diet is best adapted to maintain their nutrition. In chronic parenchymatous nephritis, although some remarkable recoveries are recorded in which the patients lived for two years or more on milk only, the majority would neither tolerate such a diet nor do well upon it.

In contracted granular kidney the chief concern is to maintain the heart, and this is best done on a mixed diet. In most affections of the heart, and in diseases like pneumonia, which throw a heavy strain upon the circulatory mechanism, it is hardly necessary to say that a pure milk diet is unsuitable, because of the large amount of fluid that must be taken.

---

